# EVA Films Loaded with Layered Double Hydroxide (LDH) Modified with Methacrylic Anion: Effect of the Nanohybrid Filler on the Photodegradation Phenomena

**DOI:** 10.3390/polym13152525

**Published:** 2021-07-30

**Authors:** Giuliana Gorrasi, Gianluca Viscusi, Giusy Curcuruto, Maria Cantarella, Alessandro Di Mauro, Paola Bernardo, Gabriele Clarizia, Andrea A. Scamporrino, Sabrina Carroccio

**Affiliations:** 1 Dipartimento di Ingegneria Industriale, Università degli Studi di Salerno, Via Giovanni Paolo II, 132, 84084 Fisciano, SA, Italy; ggorrasi@unisa.it (G.G.); gviscusi@unisa.it (G.V.); 2 Consiglio Nazionale delle Ricerche, CNR-IPCB s.s Catania, Via P. Gaifami 18, 95126 Catania, CT, Italy; giusy.curcuruto@cnr.it (G.C.); sabrinacarola.carroccio@cnr.it (S.C.); 3 Consiglio Nazionale delle Ricerche, CNR-IMM s.s Catania (Università), via Santa Sofia 64, 95123 Catania, CT, Italy; maria.cantarella@ct.infn.it (M.C.); alessandro.dimauro@ct.infn.it (A.D.M.); 4 Consiglio Nazionale delle Ricerche, CNR-ITM, via P. Bucci 17/c, 87036 Rende, CS, Italy; p.bernardo@itm.cnr.it (P.B.); g.clarizia@itm.cnr.it (G.C.)

**Keywords:** ethylene vinyl acetate copolymer, EVA nanocomposite, Layered Double Hydroxide (LDH), photo-oxidation, degradation, gas permeation, ball milling

## Abstract

The photo-oxidative studies of ethylene vinyl acetate copolymer (EVA) matrix, filled with Layered Double Hydroxide (LDH) modified with methacrylic anion (MA), were herein reported, together with gas permeation tests. The formulation of nano-hybrid LDHs was characterized using X-ray diffractometry (XRD) and thermogravimetric analysis (TGA), demonstrating the partial intercalation of the 30% of MA anion between the LDH’s galleries. The as-modified filler was introduced into an EVA matrix by mechanical milling, producing free-standing films subjected to accelerated aging. Fourier transform infrared spectroscopy (FT-IR) results suggested that the nanohybrid presence determined a stabilizing effect up to 45 days of UV irradiation, especially if compared to the EVA/LDH references for all formulated EVA hybrid nanocomposites. Conversely, the presence of nanohybrid in the matrix did not significantly change the thermal stability of EVA samples. The dispersion of modified MA-LDH in the EVA matrix produces defect-free samples in the whole range of investigated loadings. The samples show a slight decrease in gas permeability, coupled with a substantial stabilization of the original CO_2_/O_2_ selectivity, which also proves the integrity of the films after 30 days of UV irradiation.

## 1. Introduction

The primary worldwide source of energy mainly derives from fossil fuels, whose reservations are constantly decreasing. In addition, the large consumption of fossil fuels is harmful for the environment, and a great effort is needed to move from non-renewable to sustainable energy sources. Among the reliable approaches to satisfy criteria for a new green deal, the use of solar energy, being the most abundant renewable energy resource, is considered the winning strategy in different fields of application. Specifically, photovoltaics (PV) is currently the fastest growing technology, reaching the most competitive prices compared to other technologies [1]. To be a cost-effective technology, photovoltaic modules are expected to operate reliably for about 25–30 years under the interactive conditions in which they are installed [2]. It is known that photovoltaic modules work in non-controlled field conditions, that greatly influence their efficiency during the aforementioned working period. In light of this, the understanding of aging processes involved during outdoor exposure for each component of the photovoltaic module becomes of outstanding importance [2,3,4,5]. One of these elements comprises the ethylene vinyl acetate copolymer (EVA) as an encapsulation agent. The latter possesses several peculiar features, such as high transmittance, good adhesion to glass and relative weather and UV resistance, although degradation phenomena can occur for prolonged light exposure times [6,7,8,9]. Degradation of EVA involves complex chemical and physical phenomena that mainly depend on temperature, UV radiation and moisture. In this regard, since the identification of structural and physico-chemical changes involved during EVA aging can provide useful information to slow down the process, several studies have been devoted to the comprehension of polymer degradation and its stabilization. Among them, the addition of fillers by using different formulation technologies was reported as a valuable solution to extend the material lifetime in terms of light resistance and mechanical and gas barrier properties [8,10,11,12,13,14,15]. However, the selection of nanoparticles to improve the performance of polymer nanocomposites can be a difficult task. Indeed, by adding a specific filler to a specific matrix, great benefits in terms of mechanical properties can be obtained, and vice versa, the same filler can determine a depletion of UV durability. This phenomenon can be tuned, depending on the chemical composition of the nanofiller, its UV and thermal stability, its morphology and the possible interactions and/or reactions that may occur between the matrix and nanofiller [16]. In this context, Layered Double Hydroxides (LDHs) are receiving increasing interest as fillers for polymeric matrices, owing to their unique versatility. Particularly, their anion exchange capability allows to design a great variety of nano-fillers, simply by varying the intercalated species. Despite that several papers have reported the photo-oxidative behavior of polymer nanocomposites filled with LDHs [17,18,19,20,21], related studies on EVA filled with LDHs are missing in the literature. This work reports as a novelty the preparation and characterization of LDHs modified with methacrylic acid and their formulation with the EVA matrix by using a mechanical milling procedure. As stated, LDHs are able to boost the mechanical and thermal properties of several polymer matrices, although their introduction can cause serious concerns about the polymer durability. In this regard, the unusual choice to use MA as an intercalating species was made assuming that its polymerization, triggered by UV exposure, might assist in “repairing” the macromolecular structure that is subjected to unrelenting chains’ scission. Herein, the influence of the filler on degrading or stabilizing the nanocomposite materials was reported and discussed, also comparing results obtained by loading different filler concentrations and related references. Finally, transport properties versus O_2_ and CO_2_ were also evaluated, tracking the response to photo-oxidation.

## 2. Materials and Methods

### 2.1. Materials

MgCl_2_ × 6H_2_O, AlCl_3_ × 6H_2_O, NaOH and methacrylic acid (MA) were purchased from Sigma-Aldrich (Italy). Ethylene vinyl acetate (EVA) Green Flex^®^ ML 40 (14% of vinyl acetate content, Melt Flow Rate (190 °C/2.16 kg) 2.5 g/10 min) was kindly supplied by Versalis. Carbon dioxide (CO_2_) and oxygen (O_2_), used in the permeation tests, had a purity of 99.99% and were purchased from SAPIO (Italy). LDH in carbonate form (cas number: 11097-59-9) was purchased from Sigma-Aldrich (Saint Louis, MO, USA).

### 2.2. MgAl-Methacrylate (LDH-MA) Preparation by Coprecipitation Method

Fifty mL of an aqueous solution of MgCl_2_ * 6H_2_O (16.8 g, 82.8 mmol) and AlCl_3_ * 6H_2_O (10 g, 41.4 mmol) was added to fifty mL of a methacrylic sodium salt solution (5.8 g, 66.7 mmol) under stirring and nitrogen flow. The pH slowly reached the value of 9 by adding 1M NaOH. At the end, the precipitate was washed with distilled water and left in an oven at 50 °C for 24 h, under vacuum [22]. The chemical formula obtained from the elemental analysis was the following: [Mg0.65Al0.35(OH)_2_] (C_4_O_2_H_5_)0.35 * 0.7 H_2_O, with the value of the molar fraction x = MIII/(MIII + MII) of 0.35 and molecular weight of 101.59 g/mol. The amount of methacrylic anion intercalated in MgAl-MA (LDH-MA) is 30 wt% of the total weight.

### 2.3. Preparation of EVA/LDH-MA Composites

Composites based on EVA and 3, 5 and 10 wt% of LDH-MA nano-hybrid were prepared by milling LDH-methacrylate and EVA powders at room temperature in a Retsch (Germany) planetarium ball mill (model PM 100), using a cylindrical steel jar of 50 cm^3^ with 5 steel balls of 10 mm in diameter. The rotation speed used was 580 rpm and the milling time was 3 h. Films of EVA and composites, having the same thickness ≅ 100 µm, were obtained by compression molding at 150 °C, using a Carver Laboratory press, and cooled at room temperature. Films of EVA and unmodified LDH were produced using the same experimental conditions.

### 2.4. Methods

X-ray diffraction (XRD) patterns were obtained in reflection with an automatic Bruker diffractometer D8 (Karlsruhe, Germany), using nickel-filtered Cu *Kα* radiation (Kα = 1.54050 Å) and operating at 40 kV and 40 mA, with a step scan of 0.05° of 2θ and 3 s of counting time.

The photo-oxidative degradation of neat EVA and composite films was carried out on a QUV PANEL apparatus at 60 °C, with continued exposure to UV radiation up to 60 days, in the absence of water. At least two separate films were analyzed at each exposure time. The irradiance (0.68 W/m^2^) of the UV lamps has a broad band with a maximum at 340 nm (UVA 340 lamps) [23]. 

The thermogravimetric analyses (TGA) of LDHs were carried out from 30 to 800 °C at a heating rate of 10 °C/min under air flow using a TA Instrument Q500 (TA Instruments, New Castle, DE, US). The same measurements on the films submitted to photodegradation were performed under a nitrogen atmosphere at 10 °C/min, from 50 to 600 °C. Sample weights were approximately 3–6 mg. The weight loss percent and its derivate (DTG) were recorded as a function of temperature.

Fourier transform infrared (FT-IR) characterization in ATR mode was performed by a JASCO FT/IR-4700 spectrometer (average of 10 scans, at a resolution of 4 cm^−1^). 

The permeation rates of O_2_ and CO_2_ were measured at a feed pressure of 1 bar and 25 °C in a fixed volume/pressure increase apparatus (Elektro & Elektronik Service Reuter, Germany) [24]. The instrument has a high vacuum system (turbo molecular pump after a backing pump) in order to evacuate the membrane samples. A pressure transducer monitors the pressure increase due to the gas permeation in the permeate side, where the volume is calibrated. The gas permeability (*P*) is obtained from the slope of the pressure curve at steady-state conditions. In addition, the diffusion coefficient [*D*, Equation (1)] of each gas is evaluated from the gas time-lag (*θ*) [25] that is obtained by extrapolating the linear portion of the curve on the abscissa. The solubility coefficient [*S*, Equation (2)] was indirectly obtained according to the “solution-diffusion” transport model that describes the permeation of permanent gases at low pressure in dense polymeric films [26].

*D* = l^2^/6*θ* (1)

S = P/D(2)

The ideal selectivity was calculated as the ratio of the permeability values for two gases. The film thickness was calculated as the average of multiple point measurements taken with a digital micrometer (Mitutoyo).

Quantitative determination of metal ions in solution after the sequestration procedure was performed by an Inductively Coupled PlasmapMass Spectrometry (ICP/MS) Nexion 300X (Perkin Elmer Inc., Waltham, MA, USA), using the kinetic energy discrimination mode (KED) for interference suppression. Each determination was performed three times. The accuracy of the analytical procedure was confirmed by measuring a standard reference material, Nist 1640a trace element in natural water, without observing an appreciable difference. Results obtained for LDH and LDH-MA were 18.80 and 20.21 ppm, respectively.

Film transparency was determined through an ultraviolet-visible (UV-Vis) spectrophotometer UV-2401 PC Shimadzu (Kyoto, Japan). Light transmission in UV-Vis ranges (200–800 nm) was determined. A film sample (4 × 1 cm^2^) was placed into the cell of the spectrophotometer, and the transmission value at a wavelength of 600 nm was recorded. The transparency of the films was then evaluated according to Equation (3):

(3) $$\mathrm{Transparency}\;\left(\%\mathrm{Tr}\right)=\frac{\log\left(T_{600}\right)}{x}$$

where T_600_ is the % transmittance taken at 600 nm and x is the film thickness (mm). According to this equation, the lower the transparency index value is, the higher the film transparency [27,28,29].

## 3. Results

### 3.1. Materials

**Figure 1:**
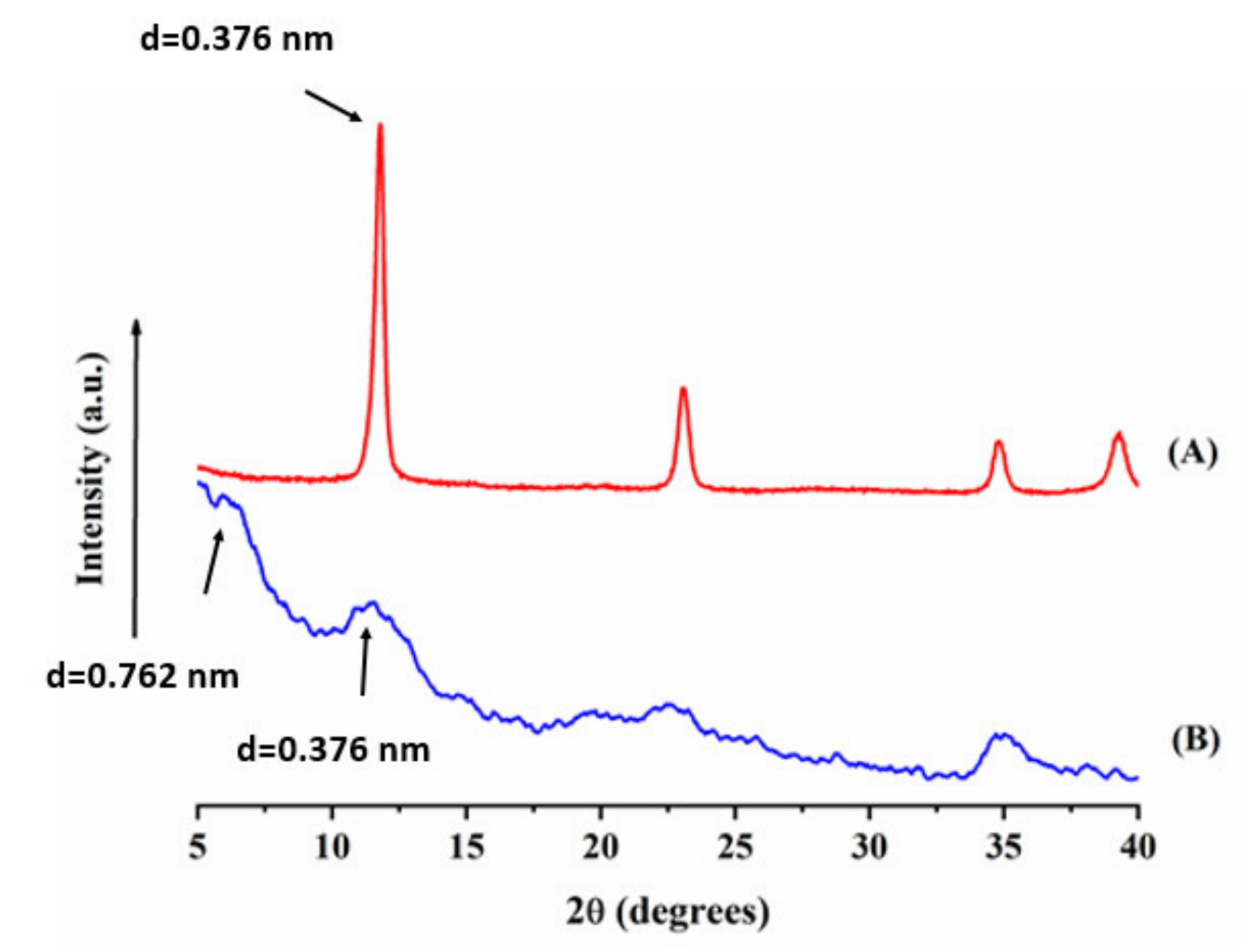
XRD of the pristine LDH with chloride anion (**A**) and the LDH modified with MA (**B**).

The as-prepared LDH-MA nanohybrid was characterized by XRD and TGA measurements. Figure 1 reports the XRD of the pristine LDH with chloride anion (A) and the LDH modified with MA (B). The pristine LDH shows the peak at 2*θ* ≅ 11.8° corresponding to the basal reflection (003) and to an interlayer distance of 0.376 nm. The XRD of LDH-MA shows that part of MA is intercalated into the pristine LDH, as evidenced by the peak at lower 2*θ* ≅ 5.8°, while part of LDH resulted as not intercalated, because of the co-presence of the peak at 2*θ* ≅ 11.8°.

**Figure 2:**
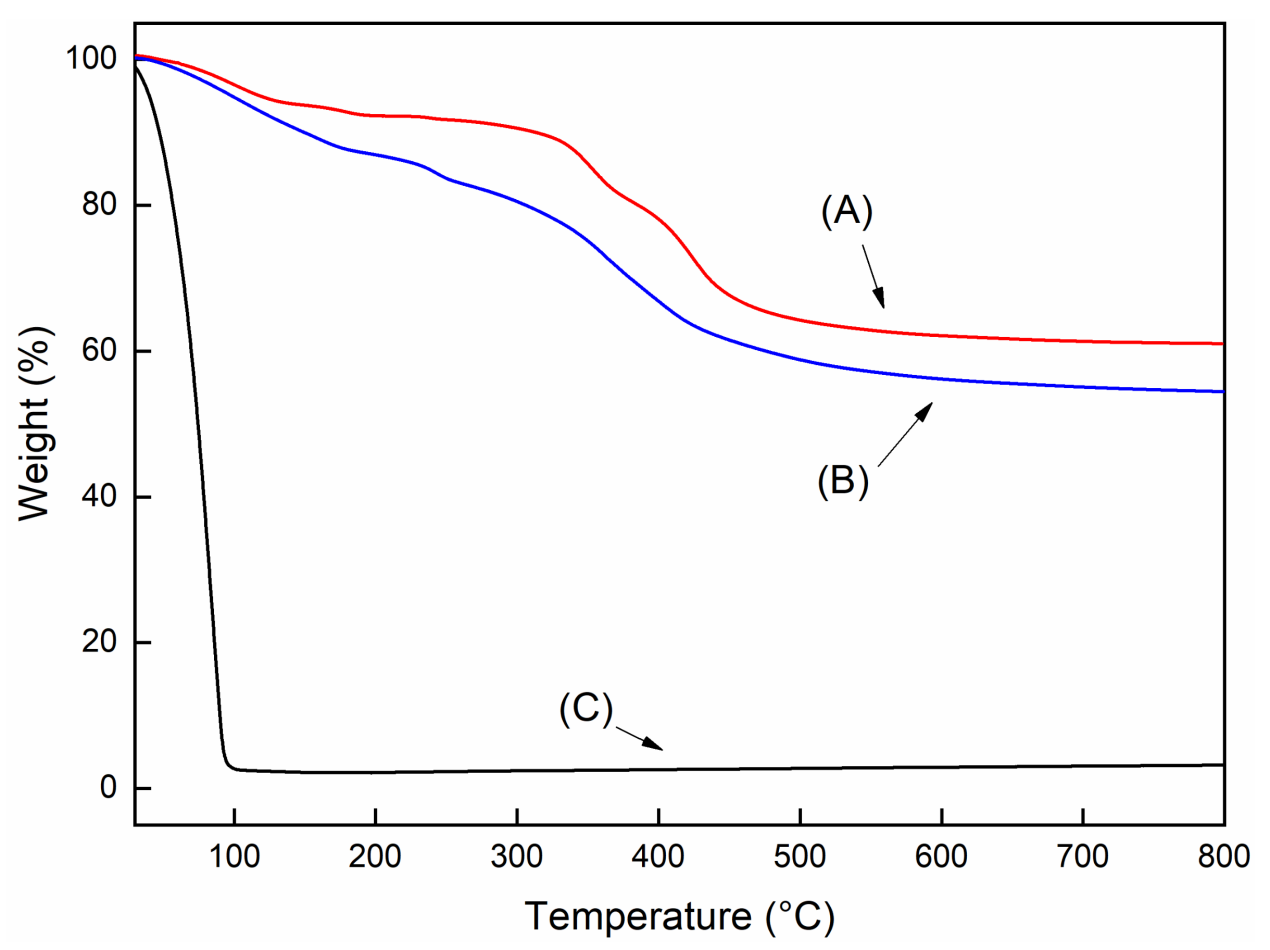
TGA of the pristine LDH with chloride anion (A), LDH modified with MA (B) and MA (C).

Figure 2 reports the TGA thermograms of the pristine LDH with chloride anion (A), the LDH modified with MA (B) and the MA (C). The first weight loss, between 100 and 140 °C, is due in both cases to the loss of intercalated water. The second weight loss, as temperature increases from 300 to 500 °C, is due to the dehydroxylation of the octahedral layers as well as the decomposition of the interlayer anion [30]. In the case of LDH-MA, the second degradation step resulted as anticipated for the presence of the intercalated MA. The methacrylic acid (MA) has a degradation temperature at about 75 °C. It is evident that the intercalation into the LDH results in a significant improvement in MA’s thermal stability, with the main thermal decomposition of the hybrid at around 350 °C. The hydroxide framework is transformed into the corresponding oxide by dehydroxylation above 400 °C. Such behavior, already found for several organic molecules intercalated into LDH layers [31], confirms that the LDH hosts constitute an interesting protection of the organic molecule, providing the possibility to incorporate thermolabile molecules even in polymers with high melting points.

### 3.2. Photodegradation of EVA and Composites 

**Scheme 1:**
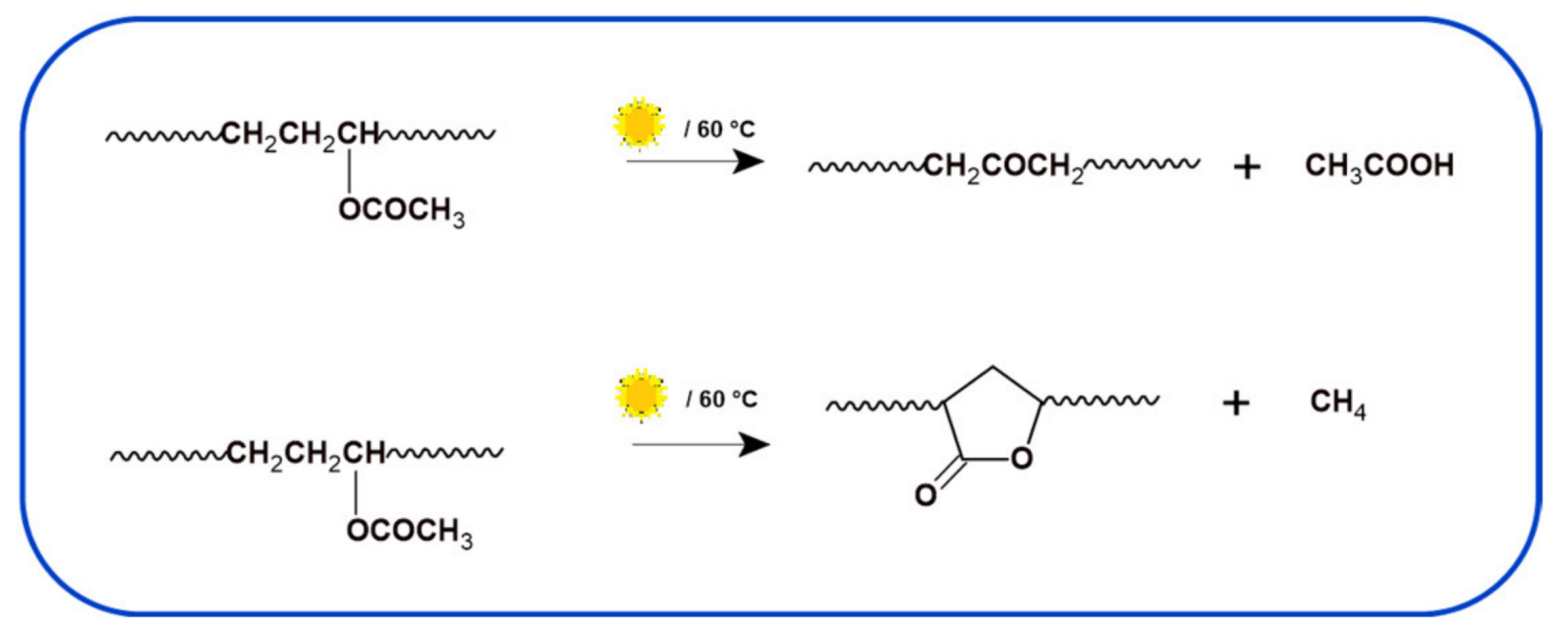
Photo-oxidative degradation with formation of acidic acid and lactone via back-biting process.

**Figure 3:**
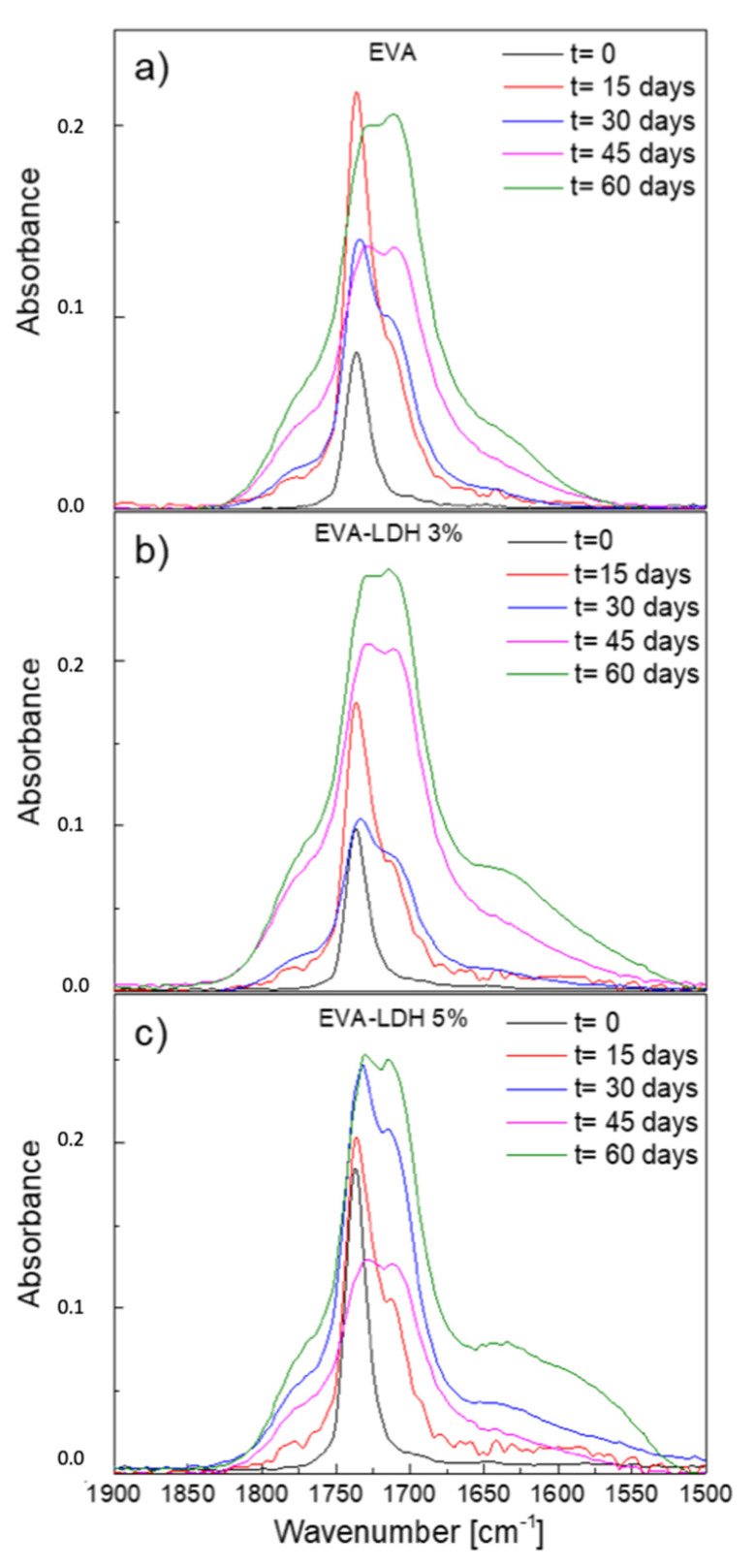
FT-IR spectra in the carbonyl region for EVA (**a**), EVA-LDH 3% (**b**) and EVA-LDH 5% (**c**) samples at different exposure times.

Samples of neat EVA, EVA + LDH and EVA + LDH−MA were subjected to accelerated aging by using a UV lamp at 340 nm for up to 60 days. To appreciate variations in chemical structure during the aging, samples were collected at different irradiation times and analyzed by TGA and FT-IR spectroscopy. Degradation of the EVA sample became detectable after 15 days; indeed, at lower exposure times, the characteristic signals remain almost unmodified (see Supplementary Figure S1). Specifically, at 30 days of photo-exposure, the EVA sample registered an increase of carbonyl signals at 1775 cm^−1^ (Figure 3a) due to the formation of lactone groups derived from UV exposure at 60 °C (Scheme 1). At longer exposure times, this peak continued to increase, confirming data reported in the literature [32,33,34,35]. By increasing the exposure time, oxidation of aliphatic groups bearing to alcohol, acid and ketone groups occurred alongside, as confirmed by the appearance of the peaks at 3484 cm^−1^ assigned to alcoholic species (Supplementary Figure S2). 

**Scheme 2:**
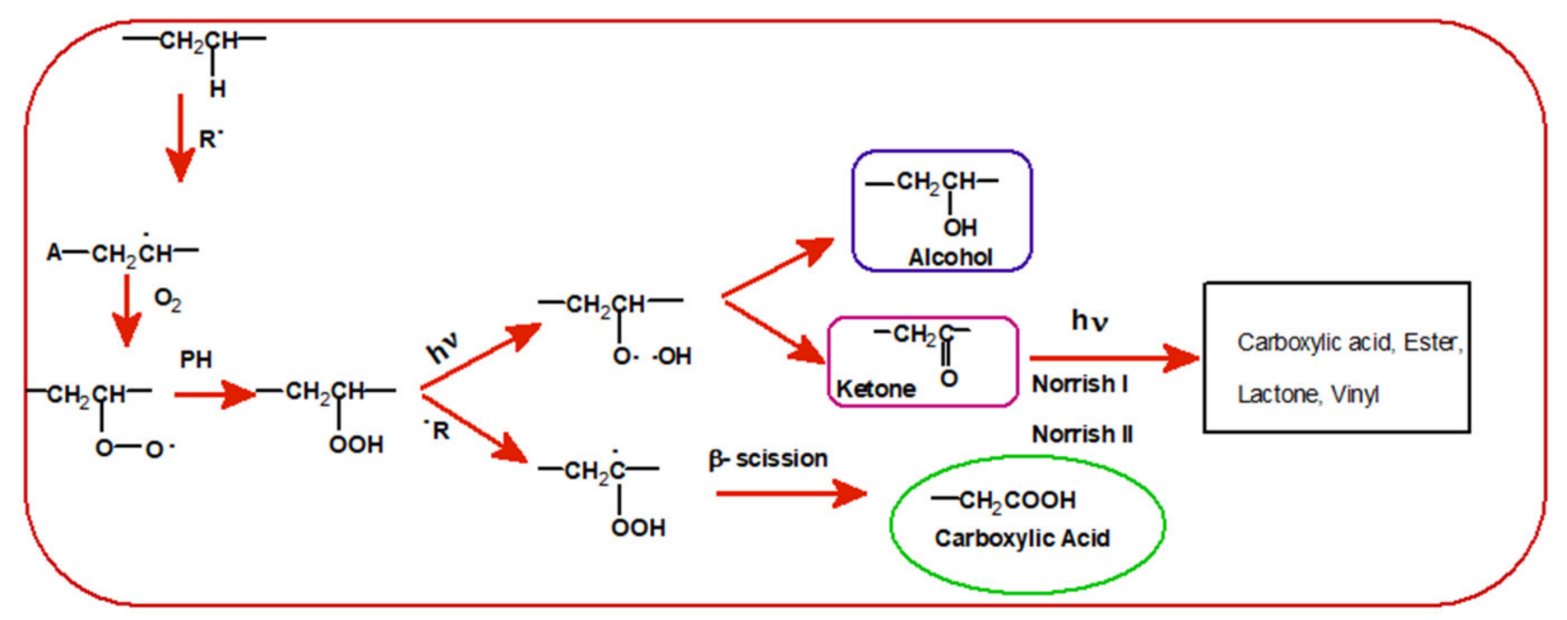
Mechanisms of photo-oxidation of PE moieties in the EVA copolymer [36].

**Scheme 3:**
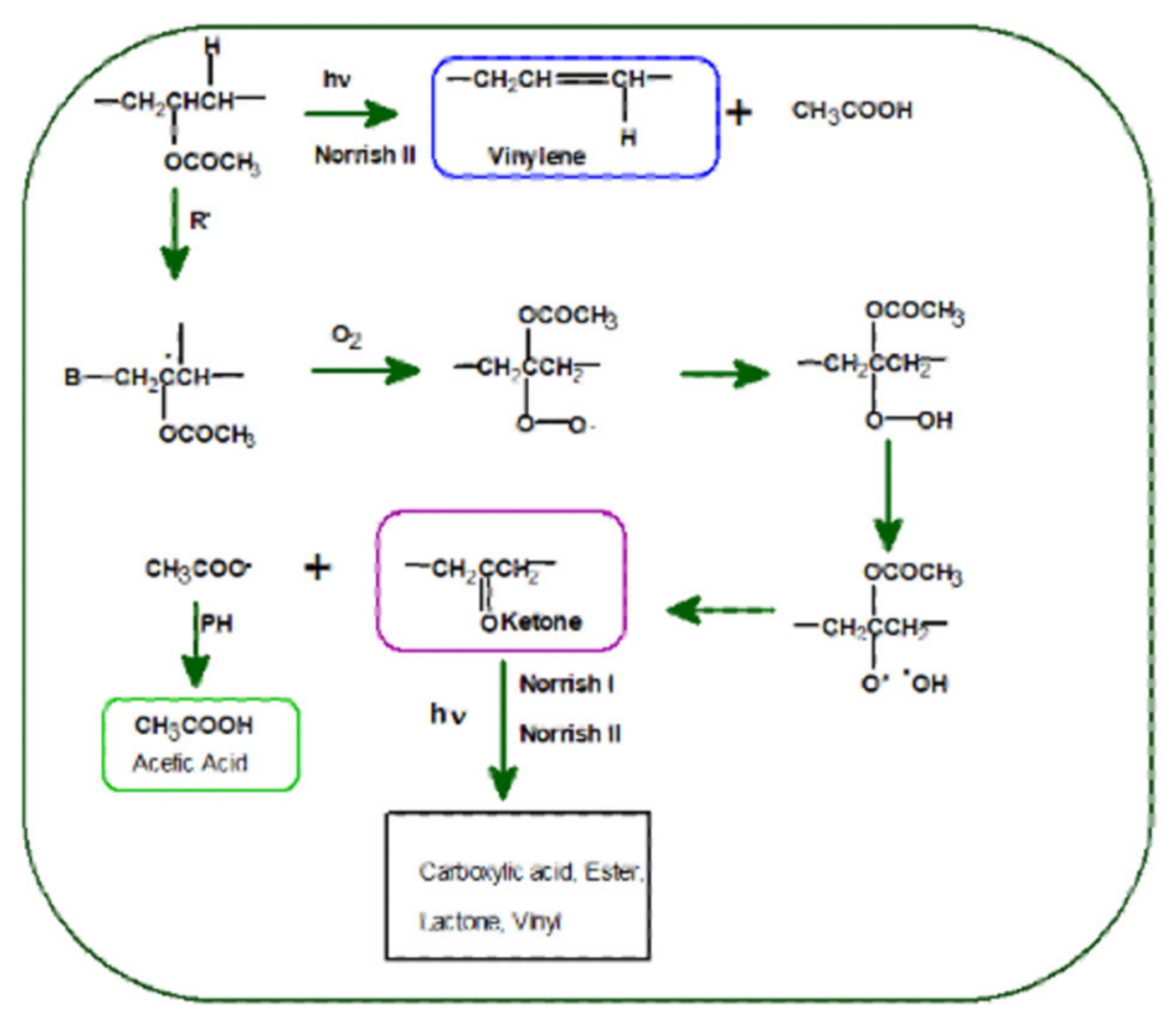
Mechanisms of photo-oxidation of VA moieties in the EVA copolymer [36].

The latter species were formed by hydrogen abstraction as well as Norrish reactions occurring in polyethylene (PE) parts (Scheme 2), and reasonably contributed to the formation of lactone, acid and ester bands. Due to the change of chemical surrounding as a function of irradiation time, the peak at 1735 cm^−1^ was shifted at lower wavelengths, whereas a shoulder at 1710 cm^−1^ assigned to the formation of acetic groups concomitantly appeared, becoming the predominant species (Scheme 1, Scheme 2 and Scheme 3). The carbonyl band related to C=O stretching is sensitive to the environment and its blueshift can be related to a strong H-bonding [36].

It is worth noticing that at higher stages of degradation, photoproducts that originated from different mechanisms were observable [36]. Particularly, photo-processes (Scheme 1) occurred in the polymer bulk where the permeation of air is restricted, whereas the exposed material surface is involved in photo-oxidative reaction pathways (Scheme 2 and Scheme 3).

As stated in the literature, polymeric matrices filled with nano-clays suffer light exposure [16,37,38]. Hence, it is not surprising that EVA filled with 5% of LDH showed an acceleration of photodegradation reactions as a function of exposure time, leading to the formation of carboxylic groups already evident for low irradiation periods (Figure 3c). As reported by Bocchini et al., LDH nanofillers can adsorb the antioxidant molecules, preventing their migration at the polymer surface and thus reducing the oxidative induction time [18].

**Figure 4:**
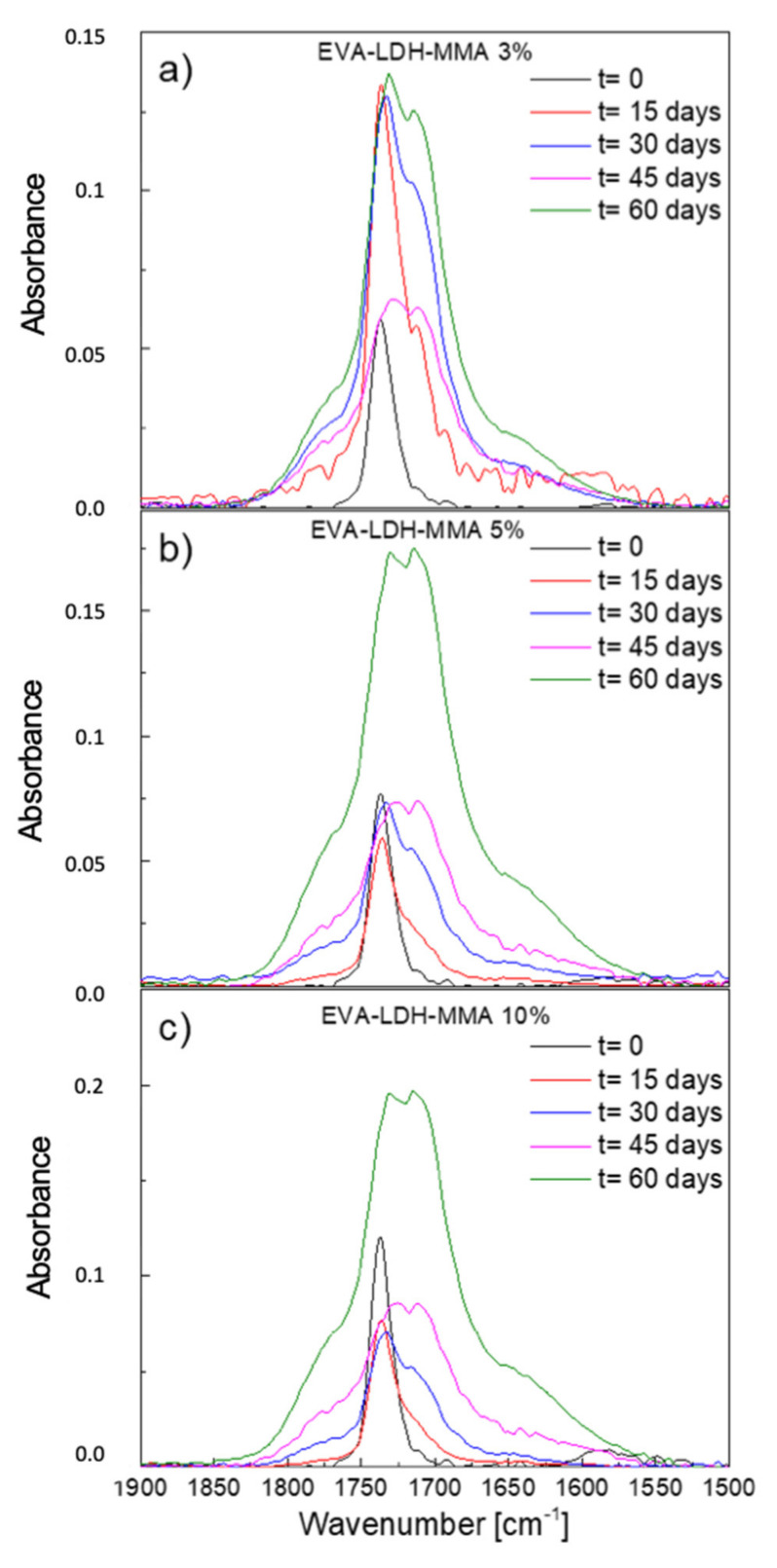
FT-IR spectra in the carbonyl region for EVA + LDH-MA 3% (**a**), EVA + LDH-MA 5% (**b**) and EVA + LDH-MA 10% (**c**) samples at different exposure times.

From the inspection of samples filled with the LDH-MA at 3%, 5% and 10%, it appears evident that the presence of LDH-MA nanohybrids changes the fate of the materials during the exposure (Figure 4). A comparison of these spectra with those recorded for the samples loaded with the not modified LDH shows a reduced incidence of the photoproducts in the films loaded with the LDH-MA nanohybrids.

**Figure 5:**
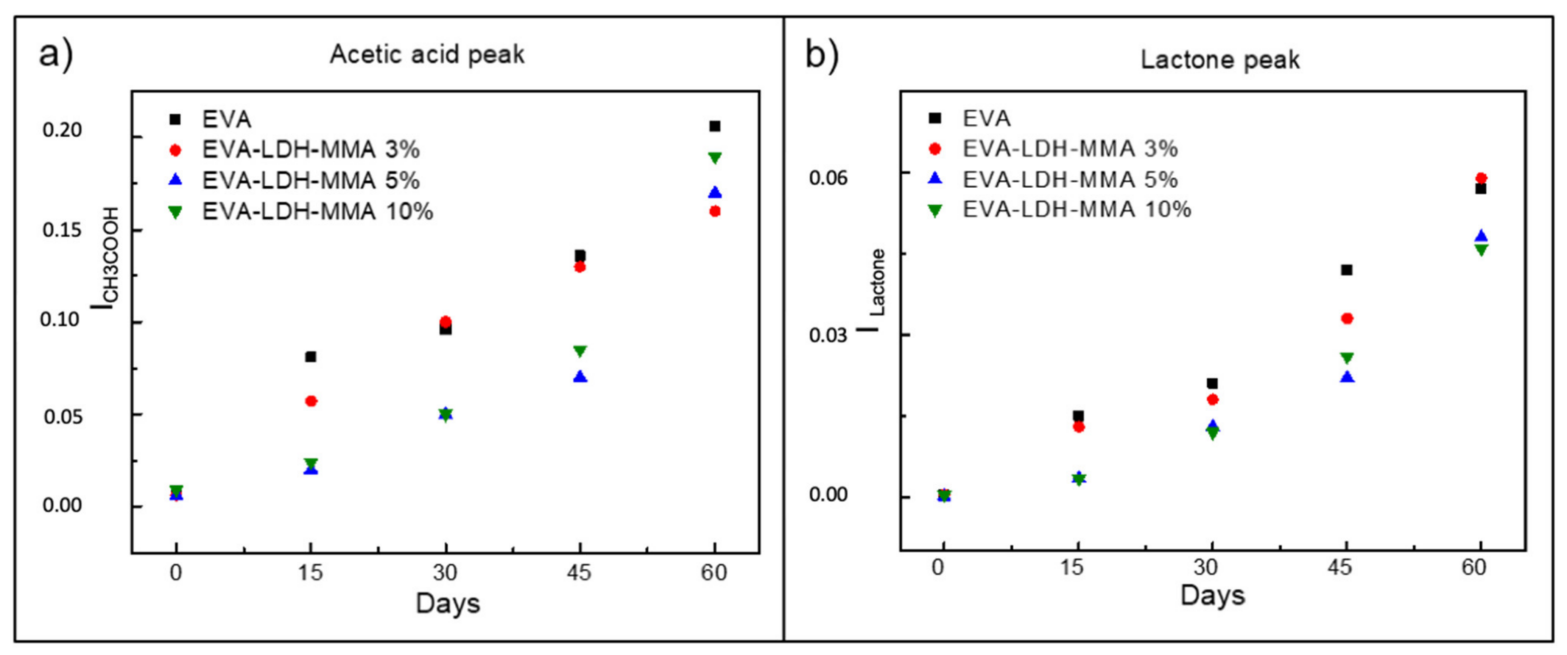
Absorbance values in FT-IR registered at different exposure times for (**a**) acetic acid and (**b**) lactone groups with the EVA samples and the samples of EVA + LDH-MA at different percentages.

Figure 5 reports the absorbance values registered for acetic acid (Figure 5a) and lactone (Figure 5b) signals as a function of the irradiation time, collected for all LDH-MA-filled samples. As observed, a stabilizing effect induced by the presence of LDH-MA over this time was shown.

Specifically, formation of lactone and acetic acid groups derived principally from the photodegradation process (Scheme 1) were drastically reduced by the presence of 5% and 10% of LDH-MA in the EVA matrix up to 45 days of exposure. 

If compared with both LDH-filled and EVA original samples, the results suggested an improved UV stabilizing effect due to the presence of nanohybrids in the matrix. It is reasonable to suppose at least two possible explanations for this experimental evidence: A different content of impurities, such as transition metal, responsible for the increase of photo-oxidation rate [18], and alternately, the embedded MA can act as sacrificial molecules by blocking radicals responsible for UV degradation and employing them in MA polymerization. To discern between the two scenarios, ICP-MS analysis of LDHs and LDH-MA were performed. As expected, a similar content of Fe ions for both samples (see Section 2.4 Methods) was obtained. In light of this, the MA polymerization seems to play a key role in preventing the photo-oxidative reactions in the EVA matrix.

**Figure 6:**
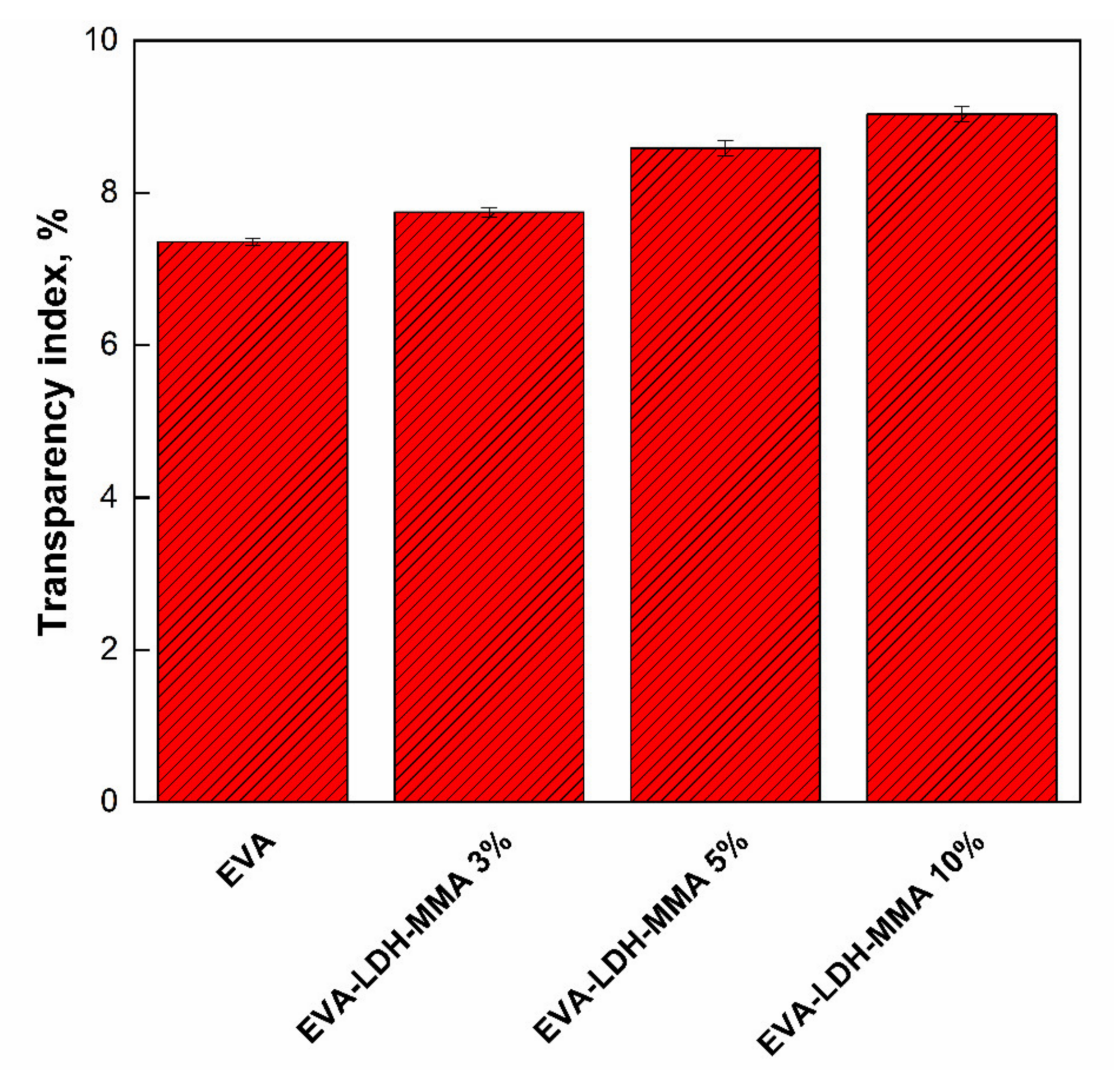
Transparency index (%) of neat EVA and EVA + LDH/MA films.

As a consequence, an increment of stabilization activity by adding more LDH-MA nanohybrid to the EVA is also expected. Samples containing 10% of nanohybrid exhibited a controversy in terms of mechanical properties. Specifically, after 45 days of photo-irradiation, the sample was extremely fragile, indicating that chain scission processes were prevalent and mostly extended for these irradiation times. Since the addition of such hybrid nano-clays can notably accomplish the photostability of the EVA matrix, suggesting its application for PV or agriculture purposes, transparency measurements were also performed. In Figure 6, the film transparency calculated by UV-Vis spectroscopy (see Section 2) revealed that such a peculiar feature, strictly required for application as an encapsulation agent or greenhouse film, was not significantly affected by the introduction of the modified LDH filler. In particular, the optical transparency was decreased by only 0.4%, 1.2% and 1.7%, with an LDH-MA content of 3%, 5% and 10% respectively, if compared to a neat EVA sample.

TGA measurements of EVA, EVA + LDH and EVA + LDH−MA films were performed to appreciate the variation of their thermal stability as a function of UV exposure time. Table 1, Table 2 and Table 3 report onset temperature of degradation (a), temperatures at maximum rate of decomposition (TMD) (b) and residual masses (c) for films based on EVA or on EVA loaded with 5% of filler. As expected, the addition of LDH fillers (Table 2, Supplementary Figure S5) increased the thermal stability of the EVA polymer for all formulations if compared to a neat EVA sample, although values underwent to a slight decrement as a function of exposure times (Supplementary Figures S4–S6, Tables S3 and S4). It is worth noting that higher LHD content triggered the formation of insoluble residue for prolonged photo-oxidation times, indicating the occurrence of crosslinking phenomena (Table 2, Supplementary Figures S4–S6, Tables S3 and S4). A different trend in thermal stability was registered for the samples containing nanohybrid at 5% (Table 3). Although LHD is added, the presence of the 30% of MA into the clay determines an inferior effect in terms of final thermal stability, resulting quite similar to that exhibited by the EVA film (Table 1). Light irradiation also induced gel formation, even if the amounts measured along the exposure time are reasonably inferior if compared with the EVA samples filled with only LHD. Similar results were obtained by analyzing EVA filled with 3% of nanohybrid.

### 3.3. Gas Permeation Tests

**Figure 7:**
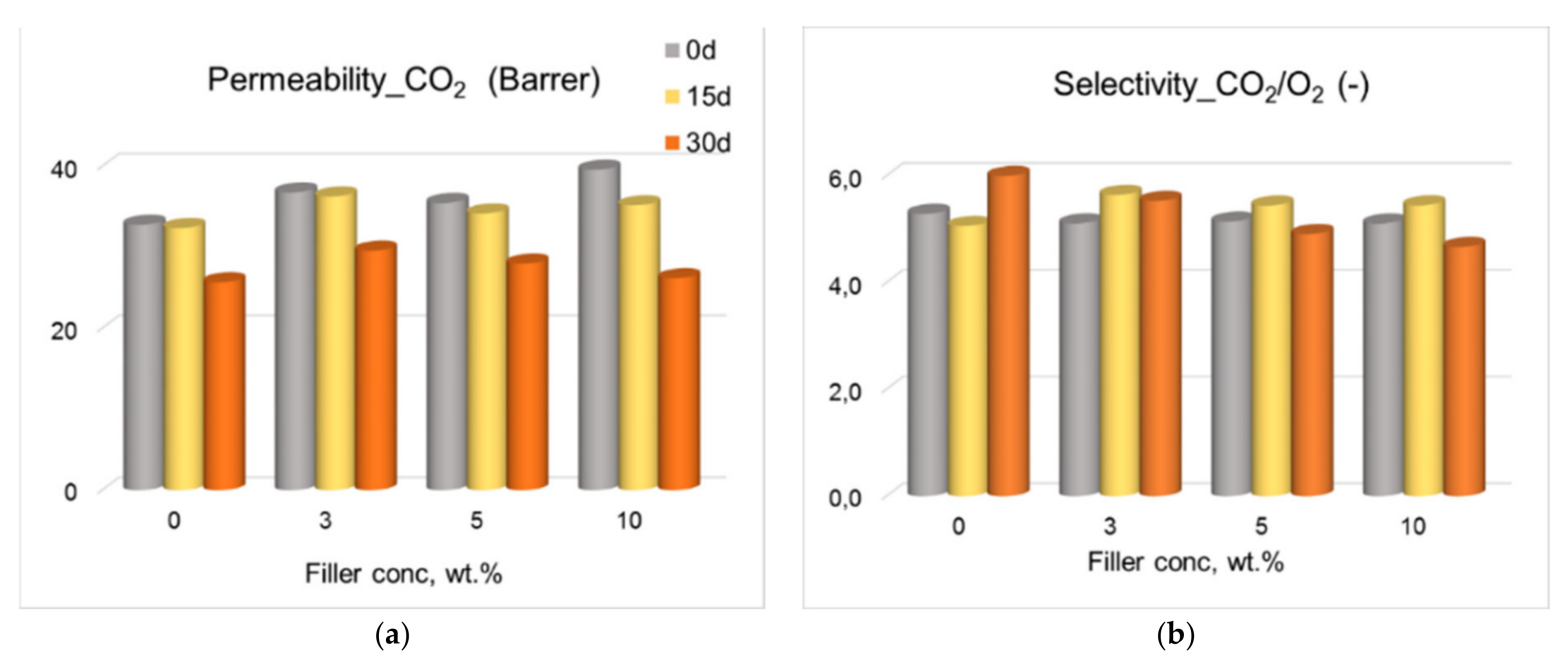
CO_2_ permeability (**a**) and CO_2_/O_2_ selectivity (**b**) for the films based on EVA with increasing loadings of LDH-MA. Film conditions: “as-prepared” (0 days) and photo-exposed (15 and 30 days). 1 Barrer = 10–10 cm^3^ (STP) cm cm^−2^ cmHg^−1^ s^−1^.

The prepared films were tested as-prepared and after the prolonged photo-oxidation treatment. The neat EVA membranes presented a CO_2_ permeability higher than O_2_. Indeed, the polar acetate group (O=C-O-) in the EVA copolymers has a preferential affinity for the polar CO_2_. The measured data on the neat EVA films are in agreement with those reported by Mousavi et al. for EVA membranes prepared by a thermal-wet-phase separation method using THF as a solvent (referred to as “EVA-28”) [39]. Interestingly, the present values are higher than those reported for membranes prepared using the thermal-phase inversion method [38]. The UV exposure reduces the gas permeability in neat EVA samples (Figure 7) and an accelerated physical aging of the films is evident at treatment times larger than 15 days.

A substantially constant permeability was observed for both gases as LDH content increased (Supplementary Figure S10). The permeability decay observed in the neat polymer was also evident in the LDH-filled films upon photo-oxidation (Supplementary Figure S10). It is coupled to a progressive decrease of CO_2_/N_2_ selectivity (Supplementary Figure S10) when the treatment time is prolonged. At higher filler content, the selectivity is further depressed, probably due to the formation of nano-defects at the polymer-filler interface resulting from the densification of the membrane matrix upon photo-oxidation.

**Figure 8:**
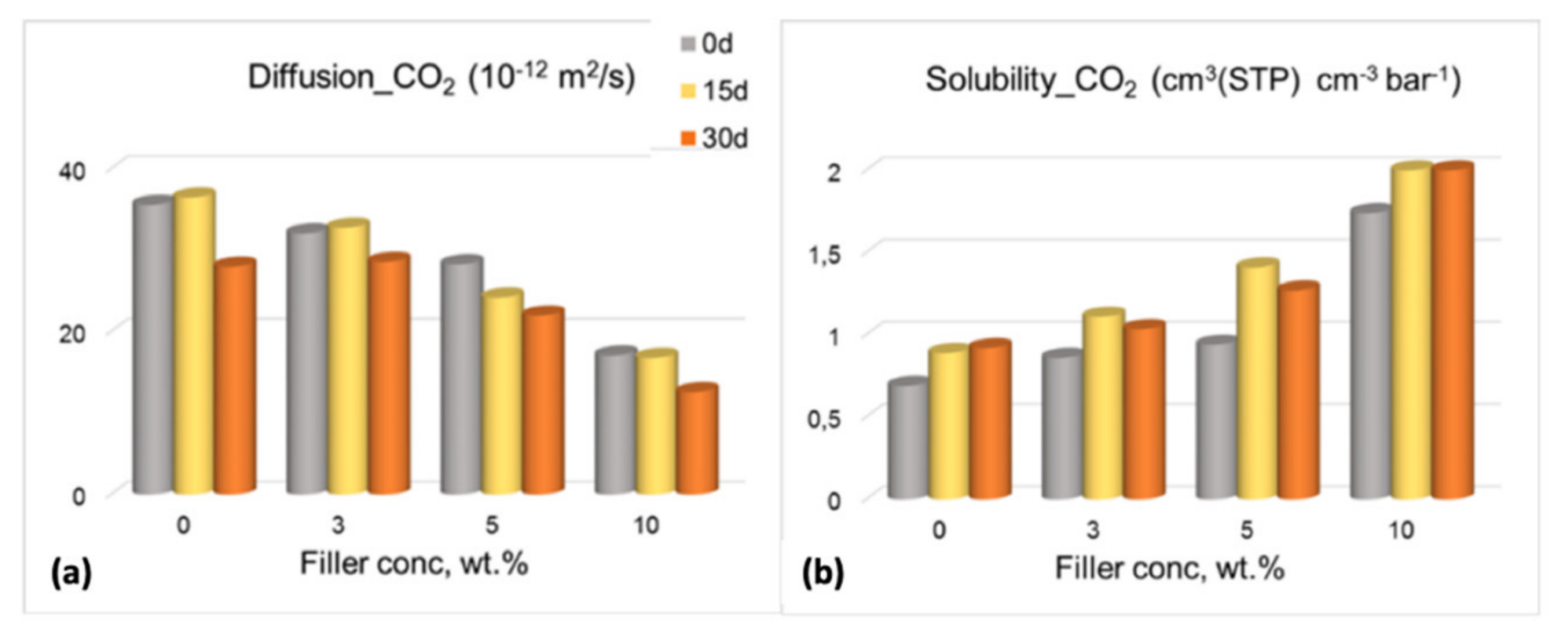
CO_2_ diffusion (**a**) and solubility (**b**) coefficients for the films based on EVA with increasing loadings of LDH-MA. Film conditions: “as-prepared” (0 days) and photo-exposed (15 and 30 days).

The gas permeation data measured in LDH-MA-loaded films evidenced the stabilizing role exerted by the modified fillers. The increase in the LDH-MA loading produced a systematically larger permeability (Figure 7) for both gases that can be attributed to the channels in the LDHs or to structural changes caused by the exfoliated lamellae within the polymer matrix. This phenomenon is coupled to a negligible change in CO_2_/N_2_ selectivity, proving the integrity of the tested films (Figure 7). The evaluation of diffusion and solubility coefficients provided more information on the nanocomposites (Figure 8). In particular, the incorporation of the modified lamellar LDHs within the EVA matrix resulted in a reduction in the diffusion coefficient owing to an increased tortuosity for the diffusion pathways. On the other hand, the calculated solubility coefficient of CO_2_ increased in the presence of the fillers, indicating a preferential affinity of the additives. However, the solubility contribution was predominant.

As already observed on the neat polymer, the photo-oxidative stress reduced the gas permeability of the films with respect to the “as-prepared” samples. Small differences were detected after a photo-exposition of 15 days, while a more pronounced permeability reduction was registered after the prolonged photo-oxidative treatment (30 days). The films remained defect-free after their photo-oxidation for irradiation times up to 30 days, and reached a similar permeability, independent of the filler amount. Indeed, the CO_2_/O_2_ selectivity remained nearly constant (ca. 5.5). A deeper analysis of the CO_2_ transport terms on the photo-oxidized films revealed, in analogy to what was observed in the as-prepared samples, a reduced gas diffusion coefficient coupled with a larger solubility upon the increase in the photo-oxidation exposure time (Figure 8). This behavior could be ascribed to crosslinking phenomena occurring on the films during the oxidation, as suggested by TGA data (Table 2 and Table 3). Song et al. demonstrated the creation of surface-densified layers for highly permeable PIM-1 membranes upon photo-oxidation treatment [40].

## 4. Conclusions

In order to take advantage of the great potential of Layered Double Hydroxides (LDHs) in formulating EVA nanocomposites without sacrificing their light durability, in this study, they were intercalated with methacrylic anion (MA) and mixed with the polymer matrix using the sustainable ball-milling process. It was demonstrated that up to 10% of loading, EVA nanohybrids maintained transparency, while their characteristic photo-oxidative processes triggered by UV irradiation were postponed at longer exposure times, allowing an increase of polymer durability. The presence of MA, that reasonably underwent to polymerization upon UV exposure, seems to play a key role in mitigating the photodegradation also triggered by the loading of nano-clays into the EVA matrix. Furthermore, the addition of LDH-MA is more effective in providing enhanced resistance to UV radiation, also noticeable in terms of gas transport properties. Indeed, their selectivity remained almost unchanged up to 30 days of exposure, whereas a slight permeability decay was observed in both neat EVA and LDH-filled samples. In general, the EVA/LDH-MA nanocomposite films demonstrated a good structural stability, since the post-treatment had no detrimental impact on their gas selectivity.

## Figures and Tables

**Table 1:** Onset temperatures of degradation, temperatures at maximum rate of decomposition and residual masses of EVA at 0, 45 and 60 days of exposure.

EVA	T_95_ (°C) ^a^	T_peak_ (°C) ^b^	Residue ^c^
t = 0	336	471	-
t = 45	326	471	-
t = 60	323	472	-

^a^ Temperature at 5 wt% of weight loss; ^b^ Temperature at the maximum derivate of weight loss; ^c^ % of residue at 600 °C.

**Table 2:** Onset temperatures of degradation, temperatures at maximum rate of decomposition and residual masses of EVA + LDH 5% at 0, 45 and 60 days of exposure.

EVA	T_95_ (°C) ^a^	T_peak_ (°C) ^b^	Residue ^c^
t = 0	337	476	0.5
t = 45	332	473	1.6
t = 60	328	479	5.3

^a^ Temperature at 5 wt% of weight loss; ^b^ Temperature at the maximum derivate of weight loss; ^c^ % of residue at 600 °C.

**Table 3:** Onset temperatures of degradation, temperatures at maximum rate of decomposition and residual masses of EVA + LDH-MA 5% at 0, 45 and 60 days of exposure.

EVA	T_95_ (°C) ^a^	T_peak_ (°C) ^b^	Residue ^c^
t = 0	336	470	1.5
t = 45	327	438	3.4
t = 60	327	465	3.3

^a^ Temperature at 5 wt% of weight loss; ^b^ Temperature at the maximum derivate of weight loss; ^c^ % of residue at 600 °C.

## Data Availability

Not applicable.

## Supplementary Materials

The following are available online at https://www.mdpi.com/article/10.3390/polym13152525/s1, Figure S1: ATR infrared spectrum of EVA at different exposure time, Figure S2: ATR infrared spectra of EVA (a), EVA-LDH 3% (b) and EVA-LDH-MMA 3% (c) as prepared, and after 45 days of exposure time, Figure S3: TGA profiles of EVA TQ as prepared, and after 45 and 60 days of exposure time, Figure S4: TGA profiles of EVA + LDH 3% as prepared, and after 45 and 60 days of exposure time, Figure S5: TGA profiles of EVA + LDH 5% as prepared, and after 45 and 60 days of exposure time, Figure S6: TGA profiles of EVA + LDH 10% as prepared, and after 45 and 60 days of exposure time, Figure S7: TGA profiles of EVA + LDH +MMA 3% as prepared, and after 45 and 60 days of exposure time, Figure S8:TGA profiles of EVA + LDH +MMA 5% as prepared, and after 45 and 60 days of exposure time, Figure S9:TGA profiles of EVA + LDH +MMA 3, 5 and 10%, Figure S10: CO_2_ Permeability and CO_2_/O_2_ selectivity for the films based on EVA with increasing loadings of LDH and photo-exposed (15 d, 30 d and 45 d).Table S1: Onset temperature of degradation, temperature at maximum rate of decomposition and residual masses of EVA+LDH+MMA 3% at 0, 45 and 60 days of exposure, Table S2: Onset temperature of degradation, temperature at maximum rate of decomposition and residual masses of EVA+LDH+MMA 10% at 0, 45 and 60 days of exposure, Table S3: Onset temperature of degradation, temperature at maximum rate of decomposition and residual masses of EVA+LDH 3% at 0, 45 and 60 days of exposure, Table S4: Onset temperature of degradation, temperature at maximum rate of decomposition and residual masses of EVA+LDH 10% at 0, 45 and 60 days of exposure, Table S5: CO_2_ Diffusion coefficient extracted from values in Figure S8.
