# Supplementary material for: EVA Films Loaded with Layered Double Hydroxide (LDH) Modified with Methacrylic Anion: Effect of the Nanohybrid Filler on the Photodegradation Phenomena
Source: Polymers (Basel). 2021 Jul 30;13(15):2525. doi: 10.3390/polym13152525 (PMC8348014; doi:10.3390/polym13152525)
Supplement: Supplementary file 1 [file polymers-13-02525-s001.zip › polymers-1308126-supplementary.pdf]

EVA films loaded with Layered Double Hydroxide (LDH) modified with methacrylic anion: effect of the nanohybrid filler on the photodegradation phenomena

Giuliana Gorrasi[1], Gianluca Viscusi[1], Giusy Curcuruto[2], Maria Cantarella[3], Alessandro Mauro[3], Paola Bernardo[4], Gabriele Clarizia[4], Andrea Scamporrino[2*], and Sabrina Carroccio[2]

[1]Dipartimento di Ingegneria Industriale, Università degli studi di Salerno, Via Giovanni Paolo II, 132, 84084 Fisciano (SA), Italy.
[2]Consiglio Nazionale delle Ricerche, CNR-IPCB s.s Catania, Via P. Gaifami 18, 95126, Italy
[3]Consiglio Nazionale delle Ricerche, CNR-IMM s.s Catania (Università), Via P. Gaifami 18, 95126, Italy
[4]Consiglio Nazionale delle Ricerche, CNR-ITM via P. Bucci 17/c, 87036 Rende, Italy

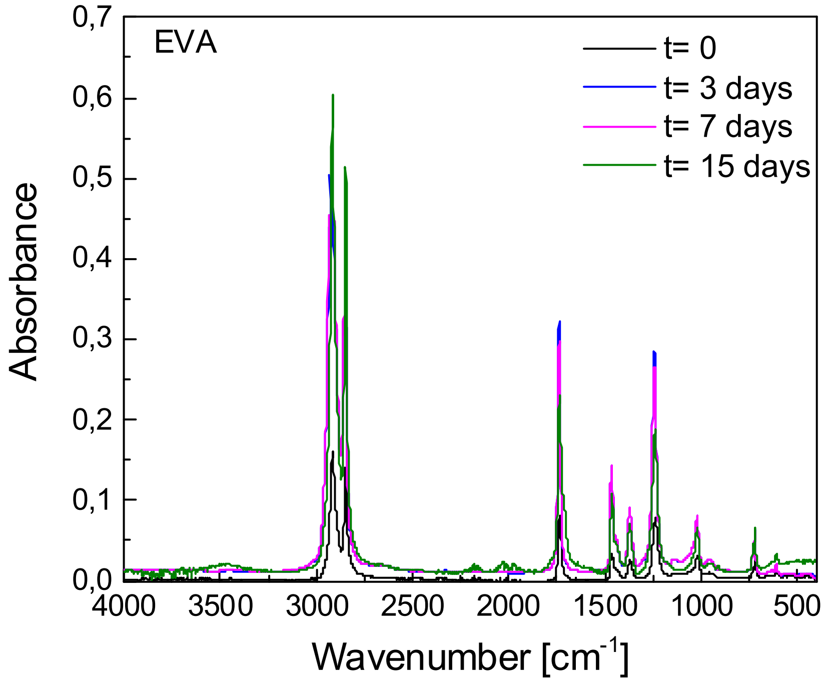

Figure S.I. 1: ATR infrared spectrum of EVA at different exposure time.

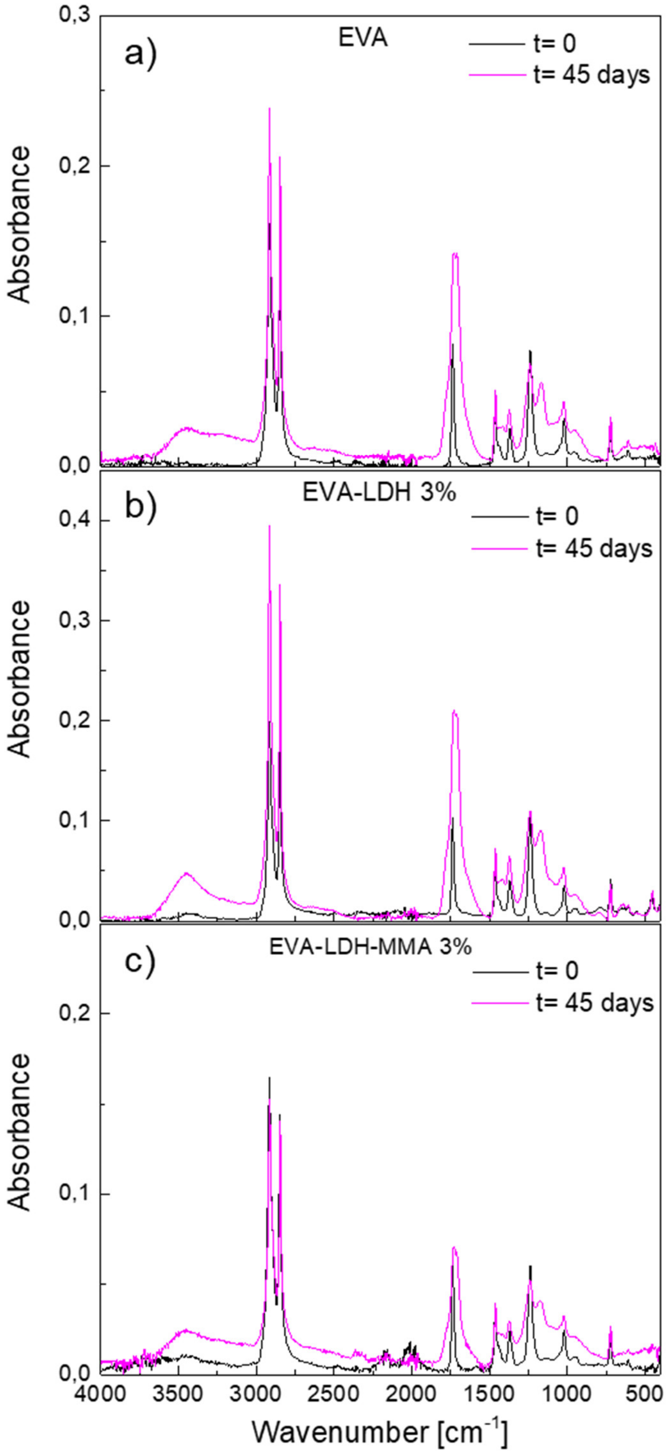

Figure S.I. 2: ATR infrared spectra of EVA (a), EVA-LDH 3% (b) and EVA-LDH-MMA 3% (c) as prepared, and after 45 days of exposure time.

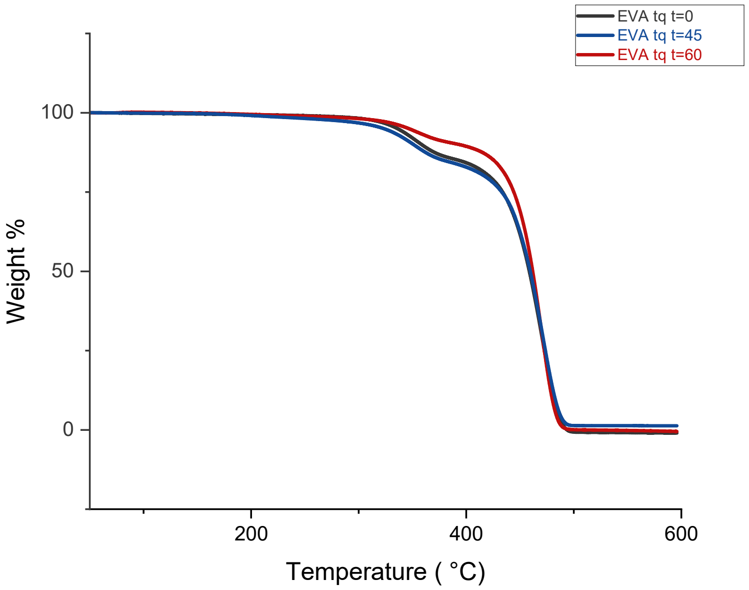

Figure S.I. 3 TGA profiles of EVA TQ as prepared, and after 45 and 60 days of exposure time.

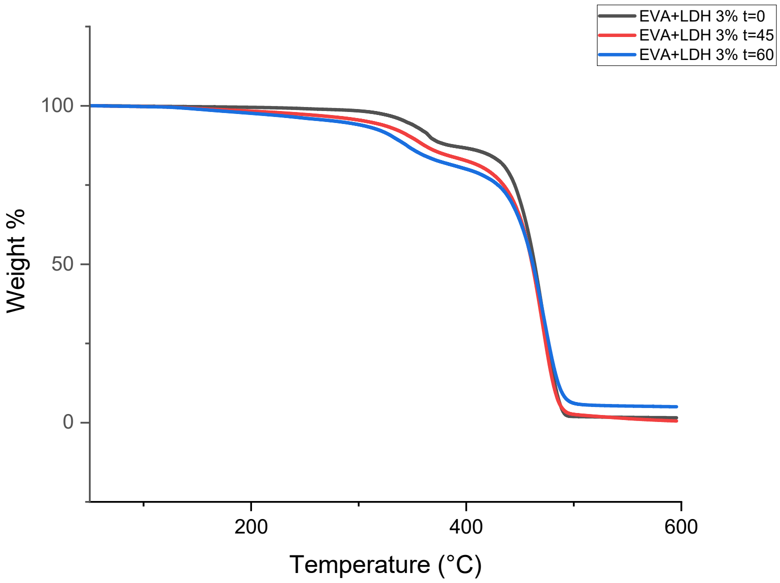

Figure S.I.:4 TGA profiles of EVA + LDH 3% as prepared, and after 45 and 60 days of exposure time.

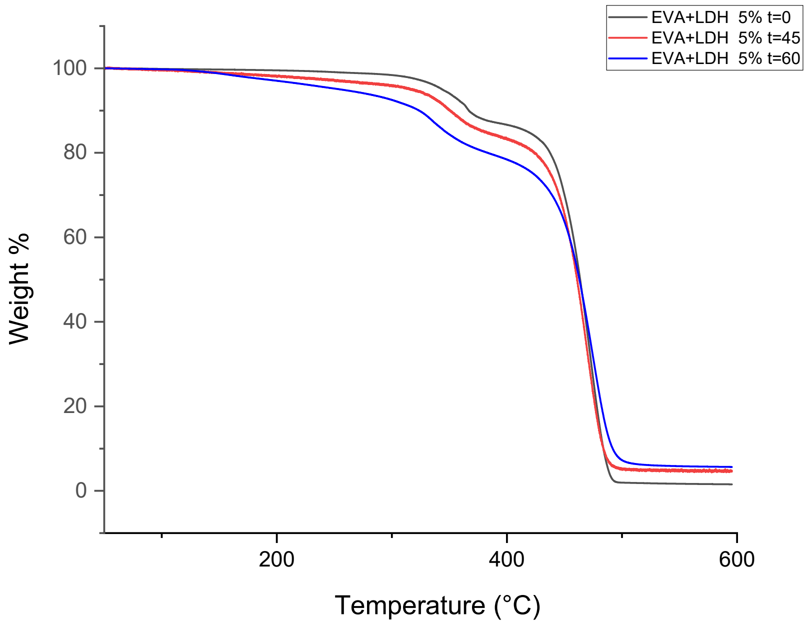

Figure S.I.:5 TGA profiles of EVA + LDH 5% as prepared, and after 45 and 60 days of exposure time.

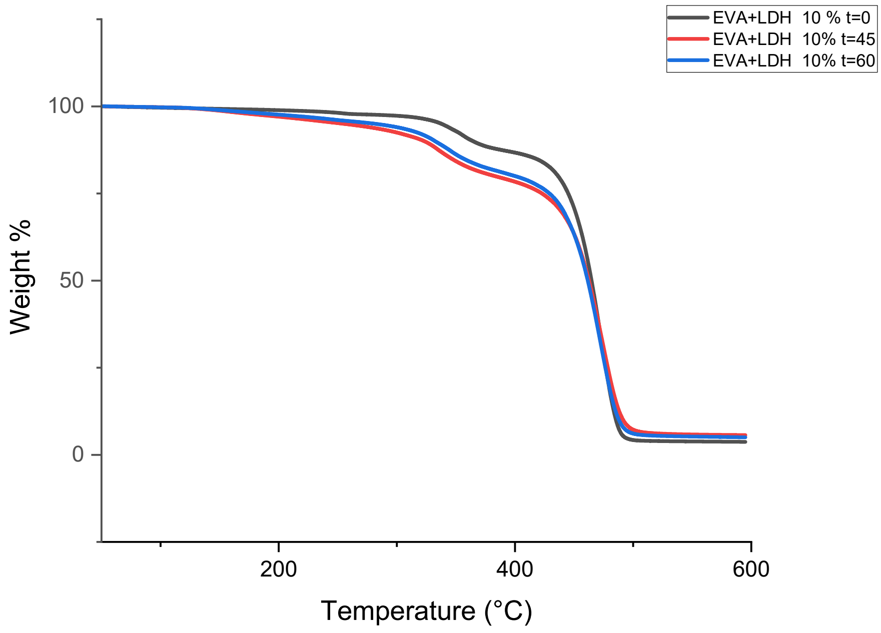

Figure S.I.:6 TGA profiles of EVA + LDH 10% as prepared, and after 45 and 60 days of exposure time.

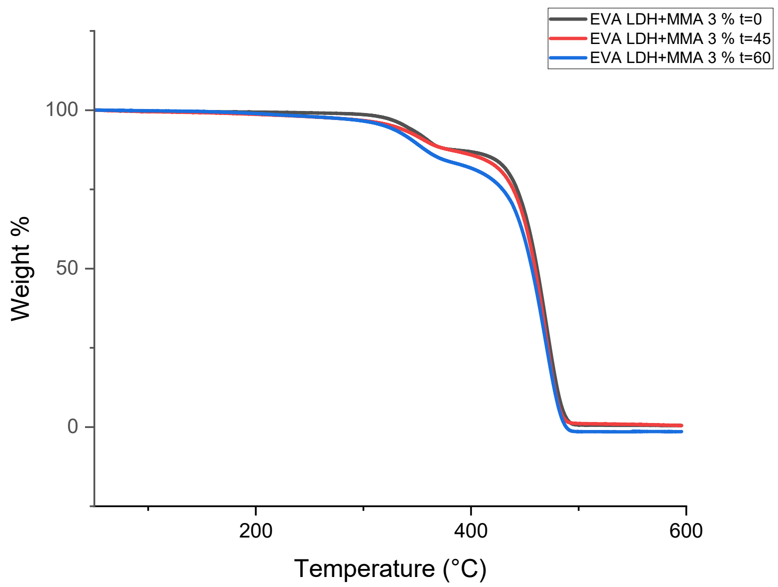

Figure S.I.:7 **TGA profiles of EVA + LDH +MMA 3% as prepared, and after 45 and 60 days of exposure time.**

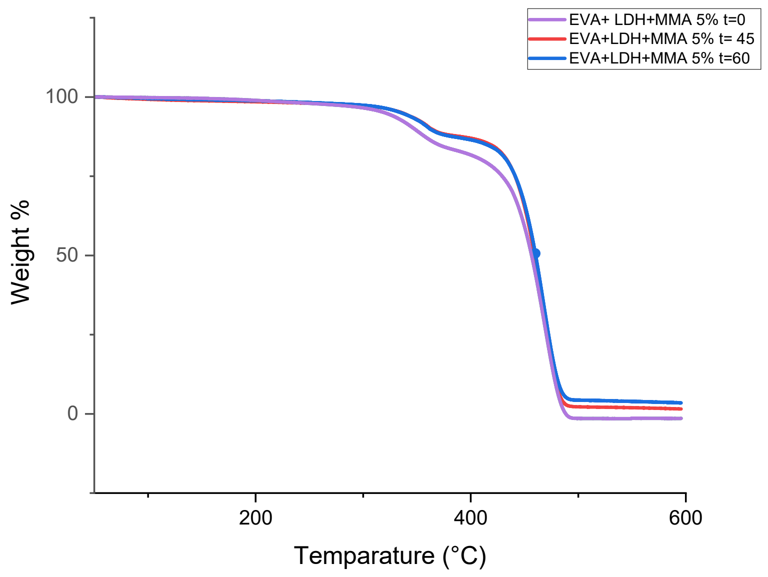

Figure S.I.:8 **TGA profiles of EVA + LDH +MMA 5% as prepared, and after 45 and 60 days of exposure time.**

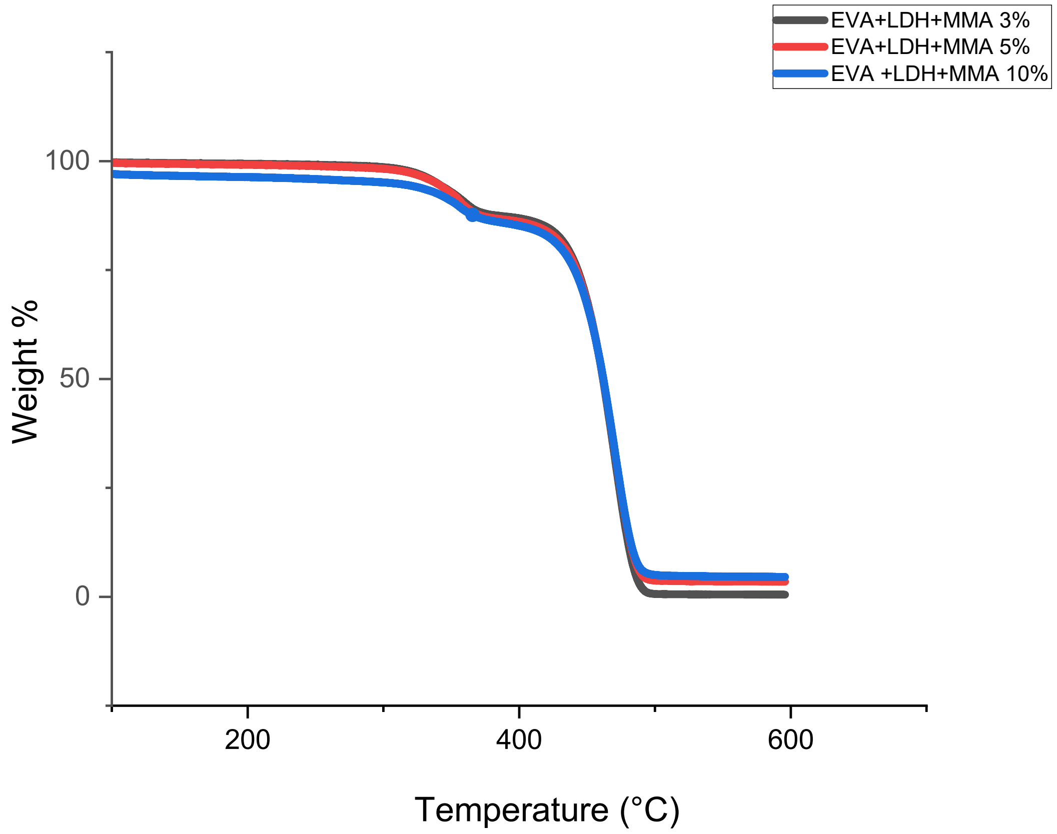

Figure S.I.:9 TGA profiles of EVA + LDH +MMA 3, 5 and 10%

TGA measurements on EVA+LDH+MMA 3%, EVA+LDH+MMA 10%, EVA+LDH 3% and EVA+LDH 10% were performed in presence of N_2 using a thermogravimetric apparatus (TA Instruments Q500) under air flow (60 mL min−1) with a heating rate of 10 °C min−1 from 50 to 600 °C. The obtained results were summarized in Tables as a follow.

Table S.I. 1: Onset temperature of degradation, temperature at maximum rate of decomposition and residual masses of EVA+LDH+MMA 3% at 0, 45 and 60 days of exposure.

Sample	*T_{95} (°C)[a]*	*T_{peak} (°C)[b]*	*Residue at T=600°C*
Eva+LDH+MMA 3% t=0	323	471	0.8
Eva+LDH+MMA 3% t=45	318	470	0.5
Eva+LDH+MMA 3% t=60	316	469	0.1

(a) Temperature at wt -5% of weight loss

(b) Temperature at the maximum derivate of weight loss

(c) % of residue at 600°C

Table S.I. 2: Onset temperature of degradation, temperature at maximum rate of decomposition and residual masses of EVA+LDH+MMA 10% at 0, 45 and 60 days of exposure.

Sample	*T_{95} (°C)*[a]	*T_{peak} (°C)*[b]	*Residue at T=600°C*
Eva+LDH+MMA 10% t=0	336	470	1.6
Eva+LDH+MMA 10% t=45	328	467	1.8
Eva+LDH+MMA 10% t=60	325	467	2.3

(a) Temperature at wt -5% of weight loss

(b) Temperature at the maximum derivate of weight loss

(c) % of residue at 600°C

Table S.I.: 3 Onset temperature of degradation, temperature at maximum rate of decomposition and residual masses of EVA+LDH 3% at 0, 45 and 60 days of exposure.

Sample	*T_{95} (°C)*[a]	*T_{peak} (°C)*[b]	*Residue at T=600°C*
Eva+LDH 3% t=0	305	473	0.8
Eva+LDH 3% t=45	299	471	0.5
Eva+LDH3% t=60	275	467	2.0

(a) Temperature at wt -5% of weight loss

(b) Temperature at the maximum derivate of weight loss

(c) % of residue at 600°C

Table S.I.: 4 Onset temperature of degradation, temperature at maximum rate of decomposition and residual masses of EVA+LDH 10% at 0, 45 and 60 days of exposure.

Sample	*T_{95} (°C)*[a]	*T_{peak} (°C)*[b]	*Residue at T=600°C*
Eva+LDH 10% t=0	338	473	3.7
Eva+LDH 10% t=45	334	474	5.6
Eva+LDH10% t=60	325	472	5.1

(a) Temperature at wt -5% of weight loss

(b) Temperature at the maximum derivate of weight loss

(c) % of residue at 600°C

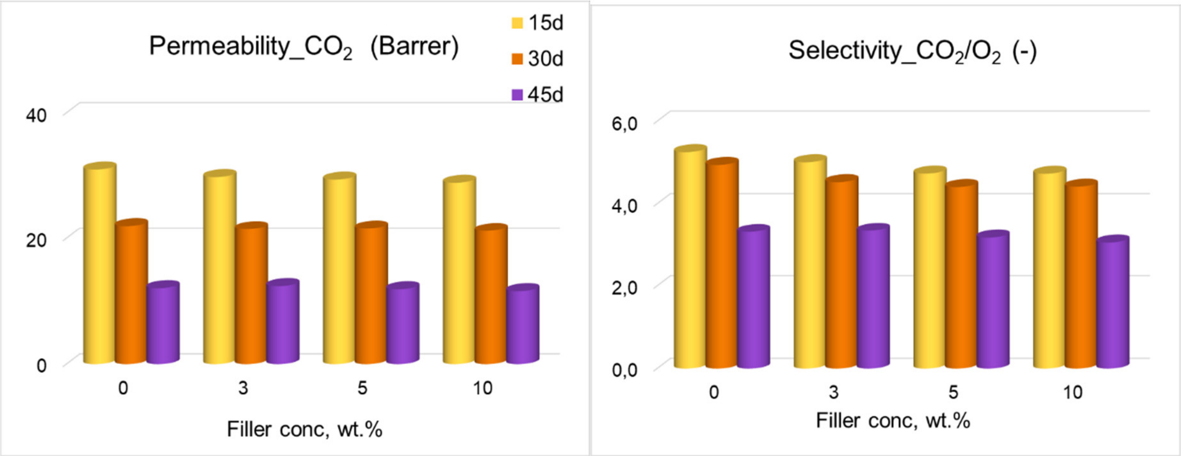

Figure S.I. 10: CO_2 Permeability and CO_2/O_2 selectivity for the films based on EVA with increasing loadings of LDH and photo-exposed (15 d, 30 d and 45 d).

Table S.I.: 5 CO_2 Diffusion coefficient extracted from values in Figures 8

Treatment time	LDH concentration, %	CO_2 Diffusion coefficient, 10^{-12} m^2/s
0 days	0	35.6
	3	32.1
	5	28.3
	10	17.1
15 days	0	36.5
	3	32.8
	5	24.2
	10	16.8
30 days	0	28.0
	3	28.6
	5	22.0
	10	12.6
